# Point-of-care Transperineal Ultrasound to Diagnose Abscess in the Emergency Department

**DOI:** 10.5811/cpcem.2019.6.43514

**Published:** 2019-09-18

**Authors:** Hamid Shokoohi, Matthew Pyle, Sarah E. Frasure, Ubah Dimbil, Ali Pourmand

**Affiliations:** * Harvard Medical School, Massachusetts General Hospital, Boston, Massachusetts; † The George Washington University School of Medicine and Health Sciences, Washington, District of Columbia

## Abstract

Perineal and rectal pain are common presentations in the emergency department (ED). In the majority of cases, clinical examination is sufficient to detect local anorectal pathologies. However, perianal and rectal abscesses and fistulas are often the primary concerns prompting diagnostic imaging in the ED. Currently, computed tomography is the preferred imaging modality. Recently, transperineal ultrasound has emerged as an optimal imaging modality for the diagnosis of perineal and perianal abscesses. We present a case in which point-of-care ultrasound accurately detected an intersphincteric abscess, and review the appropriate ultrasound technique to evaluate patients with suspected perianal and rectal abscesses.

## INTRODUCTION

Uncomplicated anorectal abscesses are typically diagnosed during clinical examination without the need for further imaging. Along with the symptoms of localized pain, mass effect, and erythematous swelling, many of these patients have a visible abscess on the external perianal skin.^1^ Infection typically arises when bacteria or fecal matter obstruct crypto-globular glands, causing either superficial or deep abscesses to form.^1^,^2^ Specifically, these abscesses arise either between the internal and external anal sphincters (intersphincteric abscesses), or externally on the skin.^3^ In deeper abscesses, such as intersphincteric abscesses, computed tomography (CT) is the most common imaging modality to detect the presence and extension of the abscess.^4^ However, CT shortcomings in the diagnosis of perirectal abscesses have been reported in multiple studies.

In a retrospective study by Caliste et al. CT missed 23% of abscesses that were confirmed with surgery.^4^ In this study, the sensitivity of CT in detecting perirectal abscesses in immunocompetent patients was 77%.^4^ Magnetic resonance imaging (MRI) and endorectal ultrasonography are alternative imaging modalities with higher accuracy. However, their use in the emergency department (ED) is limited, partly because of high cost, inconvenience to patient and physician, significant time needed to complete the exam, and lack of availability in the ED.^5^–^8^

In recent years, there has been increasing interest in transperineal ultrasound (TPUS) for multiple applications including the diagnosis of perineal and perirectal abscesses and fistulas.^9^ In the ED, a point-of-care TPUS can be employed favorably due to low cost, wide availability, lack of ionizing radiation, and minimal inconvenience and discomfort to patients. Due to these benefits, TPUS has the potential to be included in the diagnostic workup of perianal abscess and rectal discomfort as the initial imaging modality of choice in the ED. Despite these advantages, this ultrasound technique is currently underused in the ED due in part to inadequate provider familiarity with the technique.^10^ Appropriate training is essential to enable more emergency physicians and patients to reap the benefits of TPUS.^10^ This article highlights the utility and feasibility of TPUS in a patient presenting to the ED with a chief complaint of rectal pain.

## CASE REPORT

A 52-year-old male, with no past medical history, presented to the ED with a four-day history of rectal pain and generalized body pain. He reported that his rectal pain was worse with ambulation and bowel movements. He endorsed changes in bowel habits and constipation that began two days before his ED presentation. He denied fever, chills, nausea, vomiting, abdominal pain, lower extremity pain, swelling, dysuria, hematuria, and hematochezia. Of note, the patient had recently returned from Ethiopia. He was not taking any medication on a regular basis. He denied alcohol or tobacco use. His vital signs were as follows: blood pressure 159/77 millimeters of mercury, heart rate 88 beats per minute, temperature 98.4° Fahrenheit, respiratory rate 16 breaths per minute, and peripheral oxygen saturation 99% on room air.

On exam, he was with no acute distress. His abdominal exam revealed a soft, non-distended, and non-tender abdomen. A digital rectal examination revealed normal anal sphincter tone with a small fissure located in the anterior line of the anal canal, and tenderness to palpation. No hemorrhoids or rectal masses were detected in the digital rectal exam. Guaiac test was negative. The remainder of his physical exam was unremarkable. An anoscopic examination was deferred because of patient discomfort. Laboratory testing was as follows: white blood cell count 13.24 × 10^9^/liter (L) (4.5 to 11.0 × 10^9^/L), hemoglobin 14.5 grams per deciliter (g/dL) (13.5 to 17.5 g/dL), platelet count 276 × 10^3^/ microliter (μL) (150–400 × 10^3^ /uL). His comprehensive metabolic panel was unremarkable.

**CPC-EM Capsule**

What do we already know about this clinical entity?

*Although clinical examination is usually sufficient to detect anorectal pathology, computed tomography imaging is generally performed when the provider suspects a perianal or rectal abscess*.

What makes this presentation of disease reportable?

*Providers can utilize point-of-care ultrasound (POCUS) to detect perineal and perianal abscesses in the emergency department (ED) which can expedite appropriate consultation and treatment*.

What is the major learning point?

*In cases where the provider suspects an anorectal abscess POCUS may be used as a quick and accurate initial imaging study for patients in the ED*.

How might this improve emergency medicine practice?

*With appropriate training for providers, POCUS could become the preferred diagnostic imaging modality to look for perineal pathologies in the ED*.

**Image 1:**
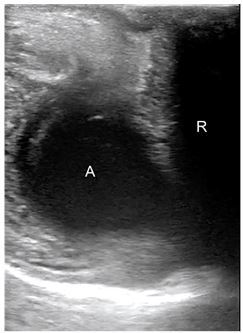
Sagittal sonographic view of the upper anal canal with space-occupying hypoechoic lesion (A) adjacent to the posterior canal and possible connection to the anal canal (R). rectal ampulla; *A*, abscess.

**Image 2:**
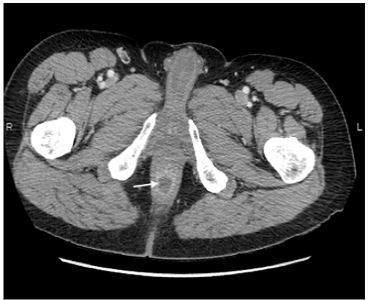
Axial computed tomography shows the intersphincteric abscess measuring 2.0 × 1.8 centimeters posterior to the anal canal (arrow).

At this juncture, we performed a point-of-care TPUS that showed a discrete intersphincteric abscess (Image 1 and Video) confirmed by subsequent CT (Image 2). Surgical consult in the ED recommended antibiotic therapy, gastrointestinal decompression, anti-inflammatory treatment, and supportive rehydration therapy. The patient was discharged home with a plan to return to the outpatient clinic for his follow-up.

The ultrasound examination was performed using SonoSite X-Porte machine (SonoSite, Bothell, WA) with 5-1 megahertz (MHz) phased array and 13-6 MHz linear transducers. Scanning was performed with the patient in the lateral decubitus position with the upper hip fully flexed. To obtain a mid-sagittal view, the phased array and linear transducers were placed on the perineum with the probe orientation marker directed toward the symphysis pubis (cranioventral). Adhesive Tegaderm transparent film was applied to cover the transducers.^11^,^12^

A coronal transperineal ultrasound of the anal canal showed a hypoechoic anal canal. A discrete, space-occupying hypoechoic perianal lesion was seen adjacent to the posterior anal canal measuring 1.8 × 1.5 centimeters (cm), with a possible connection to the posterior wall (Image 1 and Video). Color Doppler scanning showed no vascularity to the lesion; no extension to the perineal skin was detected. The possibility of perianal abscess was considered. Further scanning in transverse view was performed by obtaining images with the linear transducer directly over the external anal sphincter. This provides an image of the lumen of the anorectal canal, and surrounding soft tissue.

## DISCUSSION

TPUS has the potential to be used as the initial diagnostic imaging modality for perineal pathologies in the ED (Table). Several factors contribute to its increasing popularity, the most important being the availability of ultrasound at the bedside for a quick, convenient, and reliable scan. In this case, performing a TPUS accurately detected an intersphincteric abscess with possible connection to rectum, which was confirmed by CT as a follow-up study. Currently, there is little evidence to determine the accuracy of TPUS in the ED. However, several studies suggest high sensitivity and specificity in different clinical settings.^13^–^18^

In a recent study by Wedemeyer et al. 25 patients were evaluated to examine the use of TPUS in diagnosing perianal inflammatory disease. They compared images obtained from TPUS and MRI, and found that perianal abscesses were correctly diagnosed by TPUS in 28% (7/25) of the patients. In 24% (6/25) of the patients, MRI confirmed the presence of an abscess. However, with one patient MRI failed to correctly diagnose the abscess. TPUS did not miss any abscesses that were identified via MRI.^15^ In summary, sonography demonstrated high accuracy in the identification of perianal abscesses.

To further investigate the use of TPUS compared to CT and MRI, Rubens et al. recruited patients to examine the general anatomy and pathology of the rectal region. The authors noted that both CT and MRI lacked spatial resolution abilities, were more expensive, and did not have the real-time imaging capabilities of sonography. When examining a perirectal abscess in a 45-year-old patient with rectal pain, the author used an axial TPUS, which revealed a 2 × 2.5 cm hypoechoic collection. Another longitudinal superficial sonogram demonstrated a 4-cm collection of fluid, which later required surgical intervention. This study endorsed TPUS as the preferred imaging tool when the pathology can be visualized through this sonographic approach.^16^ Similarly, Chandwani et al. recommended point-of-care ultrasound as a diagnostic tool to confirm the presence of anorectal abscess, to visualize the spread of infection, and to properly drain the abscess in the ED.^17^

In a prospective observational study contrasting TPUS and MRI, Plaikner et al. recruited 30 patients who underwent both MRI and TPUS to compare the effectiveness and accuracy of the different modalities. In six cases out of 30 the diagnoses by MRI did not match those made by TPUS. Four cases were correctly diagnosed by TPUS, and two by MRI.^18^ While MRI still has a place in the diagnosis of perianal diseases, TPUS demonstrates at least similar efficacy. The authors concluded that TPUS should be considered whenever anorectal symptoms occur.^18^

Despite the apparent advantages of TPUS, as outlined in multiple studies (Table), TPUS is currently underused in the acute clinical setting.^14^–^18^ One potential reason is the lack of training most providers receive in imaging anorectal pathologies. It is important to consider that a substantial “learning curve” exists with TPUS because of the complex anatomy being imaged, and because of the substantial variability in patient presentation and location of rectal abscesses. In deeper abscesses the use of a high-frequency transducer is limited by its shallower penetration, which hinders visualizing deep structures. Nonetheless, Hwang et al. suggested that sonographers can become competent after completing the exam on just 12 patients.^19^ Therefore, this major limitation of TPUS may be addressed with minimal additional provider training.

## CONCLUSION

The majority of patients with anorectal pathology can be evaluated without a need for further imaging in the ED. However, in cases with potential risk of anorectal abscess, TPUS may be used as a quick and accurate initial imaging study for patients in the ED. TPUS has been proven to be a reliable and useful imaging modality in different clinical settings and can be used at the bedside by emergency physicians with proper training. It is important, however, to keep in mind that a “learning curve” exists with TPUS because of the complex perineal anatomy and limitations of high-frequency transducers to accurately detect deeper structures. Further studies to investigate the feasibility and the accuracy of this method in ED patients are recommended.

## Supplementary Information

Video. A discrete, space-occupying hypoechoic perianal lesion is seen adjacent to the posterior anal canal measuring 1.8 × 1.5 centimeters, with a possible connection to the posterior wall.

## Figures and Tables

**Table:** Perianal abscess assessment with transperineal ultrasound versus magnetic resonance imaging/computed tomography.

Study	Design	No	Findings	Conclusions
Caliste et al.^4^	Retrospective observational	113	Among patients with a confirmed perianal abscess, CT was negative in 23% of patients. The overall sensitivity of CT in the identification of perirectal abscesses was 77%.	CT lacks sensitivity in the diagnosis of perirectal abscess. This imaging tool missed nearly 25% of surgically confirmed perirectal abscesses. Therefore, another adjunct imaging modality is necessary to increase diagnostic accuracy.
Mallouhi et al.^5^	Prospective observational	87	Gray scale sonography had good accuracy in the detection and characterization of perianal inflammatory disease. For the detection of perianal abscesses, gray scale sonography sensitivity and specificity was 100% and 94%, respectively. With the addition of color Doppler sonography, accuracy in the diagnosis of perianal inflammatory disease increased.	Grey scale and color Doppler sonography have high detectability of both perianal abscesses and fistulas. When used together, these two imaging tools have increased diagnostic confidence.
Domkundwar and Shinagare^6^	Prospective observational	30	In 30 patients with confirmed anal fistulas, TPUS correctly identified 11 patients with abscesses (37%). Abscesses were hypoechoic and anechoic collections visualized on sonography.	TPUS has the potential to become the first imaging tool to diagnose patients with perianal fistulas and abscesses. Specifically, TPUS allows accurate detection of perianal abscesses. TPUS is easily available, allows real-time visualization, can be used in patients with anal stenosis, and requires no special equipment. TPUS is especially helpful when immediate diagnosis is needed, and when a more detailed imaging modality (CT and MRI) would delay diagnosis.
Stewart et al.^13^	Prospective observational	54	TPUS accurately identified perianal fistulas and abscesses in 46 patients. Specifically, TPUS diagnosed 15 abscesses correctly; 26 patients with perianal fistulas and abscesses underwent surgery following TPUS. Surgery confirmed 85% of TPUS findings.	At the Toronto General Hospital, TPUS has been implemented as the primary routine procedure to evaluate patients with any disease in the perianal region.
Chandwani et al.^17^	Case study	1	In a patient with chief complaint of rectal pain with tenesmus, point-of-care ultrasound with a 5.0 MHz curvilinear probe correctly identified a 3.6cm perianal abscess.	Using point-of-care ultrasound in the emergency department has recently increased in popularity. This imaging tool is well suited for the evaluation of patients with symptomology reflecting a potential perirectal abscess.
Plaikner et al.^18^	Prospective observational	67	36 abscesses were detected by MRI, 38 by TPUS, and 30 by surgical examination. When comparing TPUS and MRI, there was good agreement with the diagnosis of perianal abscess.	Transabdominal ultrasonography (TAS) had increased accuracy in the diagnosis of superficial rectal infections, while MRI was more suited for the identification of deeper perirectal infections.
Hwang et al.^19^	Prospective observational	43	In 43 pediatric Crohn’s patients, 18.8% of TAS examinations revealed rectal abscesses; 75% of these abscesses were associated with active fistulas.	TAS and color Doppler sonography is advantageous in the evaluation of perianal fistulas and abscesses in pediatric patients with Crohn’s disease.

*TPUS*, transperineal ultrasound; *MRI*, magnetic resonance imaging; *CT*, computed tomography; *Mhz*, megahertz; *cm*, centimeters; *TAS*, transabdominal ultrasonography.
